# The Participation Patterns of Youth with Down Syndrome

**DOI:** 10.3389/fpubh.2016.00253

**Published:** 2016-11-11

**Authors:** Megan MacDonald, Jennifer Leichtman, Phil Esposito, Nicole Cook, Dale Allen Ulrich

**Affiliations:** ^1^College of Public Health and Human Sciences, Oregon State University, Corvallis, OR, USA; ^2^Experimental Education Unit, University of Washington, Seattle, WA, USA; ^3^College of Nursing and Health Sciences, Texas Christian University, Fort Worth, TX, USA; ^4^Alcona Health Center, Oscoda, MI, USA; ^5^School of Kinesiology, University of Michigan, Ann Arbor, MI, USA

**Keywords:** Down syndrome, children and participation

## Abstract

**Purpose:**

The purpose of this paper was to investigate the participation patterns of children with Down syndrome (DS) using the construct of participation as defined by the International Classification of Functioning Disability and Health (ICF).

**Methods:**

Sixty-two children with DS were recruited between the ages of 9 and 17 years. All participants were given an interview-administered version of the Children’s Assessment of Participation and Enjoyment (CAPE) to measure participation (1).

**Results:**

Children with DS participated the most often, based on frequency, in recreational activities (*p* < 0.001); social activity types represented the greatest extension into the community based on with whom the children participated with (*p* < 0.05); finally, physical and social activities represented the greatest extension into the community geographically (*p* < 0.001). In addition, children with DS are significantly more active in activities that are informal in nature.

**Conclusion:**

Children with DS participate in a number of activities; however, the extent of their participation within these activities differs depending on the participation pattern examined. Implications for educational and community-based programs are discussed.

## Introduction

The physical, social, academic, and spiritual growth of children and adults with and without disabilities is positively influenced by active participation (2–5). Active participation is the level of participation that allows individuals to gain positive outcomes in multiple domains such as the physical, cognitive, and social (6) that contribute to personal growth and development (7). When initiated at a young age, active participation in physical activity can positively influence physical activity patterns into adolescence and adulthood (8, 9).

Increased physical activity over the life span can play a part in reducing potential health risks associated with physical inactivity later in life. Unfortunately, children with disabilities engage and participate less often than their peers without disabilities (10–12). This is particularly alarming considering the increased health risks for many children with disabilities (13, 14). Even with knowledge of health- and psychological-related benefits physical activity and active participation provide for this population (15, 16), participation patterns among children with disabilities are still largely underexplored (3, 17).

It was not until the acceptance of the International Classification of Functioning Disability and Health (ICF) model that participation was established as a construct in understanding disability (18, 19). The ICF’s model of disability suggests three levels of human function that are as follows: (1) body functions and structures, (2) activities, and (3) participation. Further, it states, an impairment at one or more of these three levels constitutes a disability (18). The nature and level of an individual’s disability can affect the level of participation that individual is capable and comfortable participating in (20).

Participation as defined by the World Health Organization (WHO) is “the nature and extent of a person’s involvement in life situations” (18). Although active participation has been linked to a healthy lifestyle, the acceptance of the ICF model identified the importance of participation within the broader context of disability (21). An individual’s involvement in these life situations may be effected differently based on the person’s disability and the needs associated with their disability (20). As a result, multiple aspects of participation should be considered when attempting to increase physical activity involvement, this may include the activity itself, other participants, where the activity is taking place, and how often participation occurs.

Down syndrome (DS) is the most common genetic cause of intellectual disability and occurs in approximately 1 in 700 live births (22). Individuals with DS are at an increased risk for obesity, osteoporosis, musculoskeletal disorders, and cardiovascular-related health problems (23). When children with DS were compared to their older siblings, they were found to be heavier and spent less time in vigorous physical activity (11, 24). Activity levels in children with DS have been found to decrease over time (25, 26), and the current participation patterns of children with DS remain under investigated (24, 27).

The lack of evidence-based research in this area warrants attention since community involvement and active lifestyles have been associated with positive health outcomes (23, 27, 28). In addition to physical health benefits, participation provides an opportunity for peer interaction in an environment that fosters social support, security, and self-esteem (29). The construct of participation, within the ICF model, aims to understand the whole person in a social context (23, 30, 31). Participation is influenced by personal, familial, and environmental factors, which all need to be considered during assessment and program planning (3).

Previous findings suggest that continuous participation is influenced by factors such as, participating with others, having fun, feeling successful, and independently completing activities (32, 33). Family values also influence the activities that children with disabilities have access to, which affects participation patterns (33). Though complex, understanding specific participation patterns for this population could have implications for educators and health professionals to help inform intervention and program needs for children with and without disabilities.

Evidence-based research about participation patterns in individuals with disabilities is limited (3, 34–36). Existing literature indicates that children with disabilities participate in informal activities, such as playground games more than formal activities, such as community-based sport programs (3). This is concerning given that formal activities have been associated with improved skills (motor skills, social skills, etc.), competencies, and social relationships (3). When the participation patterns of children with DS, autism spectrum disorders, and typical development were compared, children with typical development engaged in more social and recreational activities and more activities with friends (36). This participation disparity further exemplifies that children with DS have more limited opportunities to participate in activities that foster psychosocial health-related benefits (29).

By investigating the participation patterns of children with DS, intervention and community programs can be tailored to meet age-appropriate needs. School curricula and individual education plans (IEP’s) can be developed to support prerequisite skills needed to encourage participation in selected activities. Currently, the participation patterns of children with DS are unknown, thus the primary purpose of this study was to investigate the participation patterns of children with DS through the construct of participation as defined by the ICF.

## Materials and Methods

Participation patterns of children with DS were collected through an interview-administered version of the Children’s Assessment of Participation and Enjoyment (CAPE) (1). The CAPE was administered to children with DS in a one-on-one interview, approximately 45 min in length. The CAPE was administered during the baseline data collection for an adapted physical activity intervention (37). Two interviewers (graduate students in Kinesiology) met with all of the participants in this study as well as their caregivers to collect data. Both administrators trained together on CAPE administration and procedures. In addition to training for the CAPE, both administrators had multiple years of experience administering a variety of interview-based assessments [Autism Diagnostic Observation Schedule (ADOS), Peabody Picture Vocabulary Test (PPVT), Wechsler Abbreviated Scale of Intelligence (WASI)] to individuals with DS, intellectual disabilities, and autism spectrum disorders.

### Participants

Participants were recruited from an existing study that focused on implementing and adapting a physical activity program for children with autism or DS. A total of 62 children (28 males and 34 females) with DS were recruited through an adapted physical activity program in the Midwest region of the United States. The participants ranged in age from 9 to 17 years (mean age = 13.15, SD = 2.60). The ethnicities of the participants were Caucasian (*n* = 53, 85.5%), African American (*n* = 2, 3.2%), Hispanic American (*n* = 2, 3.2%), Asian American (*n* = 1, 1.6%), and unspecified (*n* = 4, 6.5%). All participants gave verbal and written assent, and a caregiver for each participant signed informed consent. All procedures were approved by the Institutional Review Board.

### Data Collection

The interview-administered version of the CAPE was used to measure participation (1). The CAPE is reliable and valid for children aged 6–21 years with and without disabilities. Validity and reliability of the CAPE was established using data from a study involving 427 children with physical disabilities (3). Intensity, enjoyment, and preference scores were significantly correlated with environmental, family, and child variables (*r* = 0.10–0.20), and all predictions reached statistical significance (*p* < 0.01, two tailed). Analysis showed sufficient internal consistency, test–retest reliability, and construct validity (3). Although primarily used in studies focused on children with physical disabilities, the CAPE has been used to survey individuals with DS (38). In addition, a special issue on participation specifically noted the importance of reaching out to diverse populations, such as the population of this study (39).

The CAPE consists of items (activities), and children respond based on activity participation during the past 4 months. Each item (or activity) is measured on five dimensions: diversity (whether or not the child participated in the activity), intensity (how often the child participated in the activity), with whom the child participated, where the child participated in the activity, and enjoyment of the activity. Each dimension is scored on an ordinal scale; a higher score represents a greater extension into the community, with the exception of enjoyment (see Table 1). Each activity is categorized into one of five distinct activity types: recreational, physical, social, skill-based, or self-improvement. Activities are further classified as formal or informal, based on work by Sloper and colleagues (40). Formal activities are those that require prior planning, have specific goals or rules, and have a coach, leader, or instructor. Informal activities are less structured and are often initiated by the child, such as playground play.

**Table 1:** **Dimensions proceeding diversity and ordinal scoring values**.

Diversity	Ordinal scale scoring values						
	1	2	3	4	5	6	7
**Intensity**	1 time in the past 4 months	2 times in the past 4 months	1 time a month	2–3 times a month	1 time a week	2–3 times a week	1 time a day or more
**With whom**	Alone	Family	Other relative	Friends	Others	–	–
**Where**	Home	Relative’s	Neighborhood	At school	Your community	Beyond community	–
**Enjoyment**	Not at all	Somewhat	Pretty much	Very much	Love it	–	–

Each activity was presented on a large cue card with an illustration of the activity and a phrase to describe it. The interviewer asked each question verbally. If the child responded “yes” to participation, the interviewer asked subsequent questions about the activity on each dimension (how often they participated in the activity, with whom they participated, where they participated, and how much they liked the activity). If the child answered “no” (to participation), the interviewer moved on to the next activity. Participants were accompanied by at least one parent or caregiver and were encouraged to answer questions independently. Parents occasionally provided participants with assistance when answering questions within a 4-month timeframe and to conceptualize vague questions in a more familiar context [i.e., when participants were asked the question, “do you ever participate in school clubs?” An example of a parent creating a more familiar context might be “what days do you go to reading club?” or “what do you do with Ms. Smith (an instructor for a specific club)”?]. Responses were recorded by the interviewer on a summary score sheet. A subset of 15 participants were interviewed twice (7 females, 8 males, mean age = 13.3, SD = 2.3) within a 3-week time period to estimate the test–retest reliability of the CAPE for youth with DS.

### Data Analysis

All data were analyzed using PASW Statistics (18.0) for Windows. A frequency analysis was conducted on the activities within each activity type, based on participation. Differences in participation across activity types (recreational, active physical, social, skill-based, and self-improvement) were determined by computing the average activity type score for each dimension (diversity, intensity, with whom, where, and enjoyment). A Wilk’s Lambda multivariate analysis was performed, to account for the within- and between-subject factors, on the mean activity type score within each of the five dimensions to verify whether or not significant differences existed by activity type within each dimension. When significant differences were found, a Bonferroni *post hoc* pairwise comparison of activity types was conducted for each dimension. To compare formal and informal activity participation, the overall diversity and intensity scores within each domain were compared using a chi square test.

Test–retest reliability was determined using the overall (total) scores for each of the five dimensions. The overall scores for diversity, intensity, with whom, where, and enjoyment were compared over two administration occasions. All scores were calculated as indicated by the scoring procedures in the CAPE manual (1). The overall diversity score was calculated by summing the diversity score across all 55 items. The overall intensity score was calculated by summing the intensity score across items and dividing by the total number of items (55). The overall with whom, where, and enjoyment scores were calculated by summing the score and dividing by the overall diversity score, which accounted for the number of activities that the child participated in.

## Results

A frequency analysis found the most common activities based on activity type; these results can be found in Table 2.

**Table 2:** **All activities categorized by activity type in order of frequency**.

No. of participants	Recreational activities
62	Watching TV or a rented movie
60	Playing board or card games
58	Doing crafts, drawing, or coloring
57	Playing computer or video games
56	Doing pretend or imaginary play
54	Playing with things or toys
54	Going for a walk or a hike
51	Playing with pets
51	Playing on equipment
48	Doing puzzles
47	Taking care of a pet
43	Collecting things

	**Physical activities**

47	Playing games
43	Doing snow sports
40	Doing individual physical activities
37	Playing non-team sports
37	Bicycling, in-line skating, or skateboarding
37	Doing team sports
27	Doing water sports
23	Gardening
19	Fishing
16	Racing or track and field
10	Participating in school clubs
9	Doing a paid job
4	Doing martial arts

	**Social activities**

60	Listening to music
59	Talking on the phone
57	Going to a party
57	Visiting
57	Making food
57	Hanging out
56	Entertaining others
55	Going to the movies
45	Going to a live event
36	Going on a full-day outing

	**Skill-based activities**

55	Dancing
39	Playing a musical instrument
31	Participating in community organizations
30	Swimming
15	Learning to dance
11	Taking music lessons
10	Learning to sing (choir or individual lessons)
10	Horseback riding
6	Taking art lessons
5	Doing gymnastics

	**Self-improvement activities**

61	Shopping
60	Reading
59	Doing a chore
55	Doing homework
50	Going to the public library
49	Doing a religious activity
40	Writing letters
31	Writing a story
20	Doing volunteer work
16	Getting extra help for schoolwork from a tutor

### Dimension Scores

A Wilk’s Lambda multivariate analysis was performed on the five activity types for each dimension: diversity, intensity, with whom, where, and enjoyment (Table 3). Significant *post hoc* activity type differences (*p* ≤ 0.05) are indicated by superscript in Table 4.

**Table 3:** **Group differences in activity type by dimension**.

Dimension	Welch (df1, df2)	*p* value
Diversity	187.92 (4, 151.42)	<0.001
Intensity	17.28 (4, 148.19)	<0.001
With whom	22.48 (4, 148.23)	<0.001
Where	31.05 (4, 147.88)	<0.001
Enjoyment	9.20 (4, 147.66)	<0.001

**Table 4:** **Diversity sum and intensity mean score by activity type**.

	Recreational	Physical	Social	Skill-based	Self-improvement
Diversity	10.34^a^	5.63^b^	8.79^c^	3.42^d^	7.11^e^
Intensity	5.12^a^	3.48^b^	4.07^bc^	3.78^b^	4.50^ac^

*Post hoc significant differences are denoted by different superscripts in each row*.

Within the diversity dimension, recreational activities were participated in significantly more than physical (*p* < 0.001), social (*p* < 0.001), skill-based (*p* < 0.001), and self-improvement activities (*p* < 0.001). Physical activities were participated in significantly more than skill-based (*p* < 0.001) and significantly less than social (*p* < 0.001) and self-improvement activities (*p* < 0.001). Social activities were participated in significantly more than skill-based (*p* < 0.001) and self-improvement activities (*p* < 0.001). Self-improvement activities were participated in significantly more than skill-based activities (*p* < 0.001).

Within the intensity dimension, *post hoc* pairwise comparisons showed that recreational activities were participated in significantly more often than physical (*p* < 0.001), social (*p* < 0.001), and skill-based activities (*p* < 0.001). Self-improvement activities were participated in significantly more often than physical (*p* < 0.001) and skill-based activities (*p* < 0.001).

Within the other dimensions, fewer *post hoc* differences were found (Bonferroni). In the dimension of with whom, participants had a significantly greater extension into the community when they participated in social activities compared to all other activity types, recreational (*p* < 0.001), physical (*p* < 0.05), skill-based (*p* < 0.01), and self-improvement (*p* < 0.001). Within the dimension of where, participants were significantly less geographically integrated into the community when they participated in recreational activities compared to physical (*p* < 0.001), social (*p* < 0.001), skill-based (*p* < 0.01), and self-improvement activities (*p* < 0.001). Within the dimension of enjoyment, two significant differences were found. Children with DS enjoyed social activities significantly more than physical (*p* < 0.001), skill-based (*p* < 0.001), and self-improvement activities (*p* < 0.001). They also enjoyed recreational activities more than physical (*p* < 0.001) and skill-based (*p* < 0.001) activities.

This sample of children participated in a higher proportion of informal activities compared to formal activities (chi square = 343.211, *p* < 0.001).

#### Test–Retest Reliability of the CAPE for Children with DS

The test–retest reliability of the CAPE resulted in the following moderate intraclass correlation scores for each of the overall dimension scores of diversity, intensity, with whom, where, and enjoyment: overall diversity *R* = 0.67, overall intensity *R* = 0.69, overall with whom *R* = 0.58, overall where *R* = 0.91, and overall enjoyment *R* = 0.80. These results demonstrate a moderate-to-high test–retest reliability.

## Discussion

Children with DS participate in all activity types represented in the CAPE, which include recreational, physical, social, skill-based, and self-improvement activities. The participants in this study engaged the most often in recreational activities followed by social, self-improvement, physical, and skill-based activities (see Table 4). Understanding the participation patterns of children with DS allows for educational and community-based programs to be aimed at age-appropriate preferences with the intent of achieving balanced participation. Understanding what activities individuals with DS participate in along with where and whom they are participating with provides an initial step in exploring their motivations and impediments.

Recreational activities were participated in the most among children with DS (the most common activities for recreational activities and other activity types are listed in Table 2). Activities within this category included playing board games, watching TV, playing computer and video games, crafts, drawing, or coloring. The results of this study support previous work, which indicate that children with DS lead physically inactive lifestyles (24, 41). To that end, the least participation occurred in the active physical and skill-based activity types – it is noteworthy that these activity types consisted of many physical activities (see Table 2) (1).

Although this study was not aimed solely at understanding physical activity participation, there is a consistent trend in the data favoring physical inactivity for children with DS. Given the health-related concerns facing children with DS as they age, such as increased risk for obesity, osteoporosis, musculoskeletal disorders, and cardiovascular related health problems (23), it is important to embed active physical and/or skill-based activity types in educational and community-based programs. Educators and health professionals should work toward providing balanced activities that include the children’s activity preferences without neglecting other priorities, such as physical activity (42).

The CAPE measures participation on a social and geographic continuum through the dimensions of “with whom” and “where.” Lower scores in these dimensions are reflective of more solitary activity (with whom) with a closer proximity to the home (where), and higher scores reflect more engagement within the community on a social and geographic continuum (1). Based on the results within these dimensions, youth with DS extend their social and geographic network the most when they participate in the active physical and social activity types. In contrast, social and geographic networks extend the least when they participate in the recreational activity type. Based on the results of this study, each activity type has unique benefits focused on different aspects of participation. This information can guide programs and help educators and community programmers to create activities based on the various needs of the program and the needs of the children.

Similar to previous studies on the participation patterns of children with disabilities, we found that children with DS prefer activities with an informal structure. King et al. (34) found that as children with physical disabilities get older, participation in recreational activities declines, and participation in social activities increases. This result is not surprising given that meaningful participation is impacted by enjoyment, and children’s interests change as they get older (33). As children get older, often the availability of resources and supervision needs decrease, understanding this impact on participation needs is important (43).

A combination of preferences and activity priority should be taken into consideration for program development. Within this study, many recreational activities were participated in frequently, while enjoyable, these activities provide little physical activity. The health-related concerns of children with DS have direct relationships to physical inactivity making physical activity a priority (42). Educational and community-based programs may be better informed through balance (a variety of activity types), preference (activity types that children enjoy participating in), and priority (activity types that focus on priority based on appropriate assessment).

For example, a child who has a priority of forming friendships should be encouraged to participate in social activities. While a child with a priority of being more physically active should focus on physical and skill-based activities. Targeting educational and community-based programs to increase participation for individuals with DS may make a significant contribution to improving their health and well-being (43). Choosing activity types targeted at priorities and preferences can allow children to participate in a balance of activities, including activities with direct health-related benefits and activities with psychosocial-related benefits such as improved social support, security, and self-esteem (29).

Understanding these participation patterns helps researchers, clinicians, interventionists, and educators better prepare for program needs that help to enhance the overall community participation for children and youth with DS. Previous research suggests a behavior phenotype for individuals with DS to engage in specific activities (44). Understanding these activities along with the less popular activities could be beneficial for practitioners working with individuals with DS. Many of the participants in our study reported deficits in the areas of formal, physical, and skill-based activity participation. These deficits could indicate a need for increased school- and community-based programs in these areas.

There are several logical next steps to extend this research. Future research can seek to link motivation to activity participation. In addition to motivation, future researchers might seek to examine potential social and motor skills necessary for successful participation. For example, adults with DS often report difficulty in finding someone to be active with (45) in addition to previous research indicating delays achieving motor milestones (46).

### Limitations

The test–retest reliability of the CAPE shows moderate-to-high levels of consistency. The small subsample used to test reliability could have been larger and produced more normal and stable measures of variability and helped improve reliability. There were some additional factors in administering this tool to youth with DS that need to be considered. Previous research suggests that CAPE administration takes approximately 30–45 min to complete (1). During this study, the approximate administration time of the CAPE for youth with DS ranged from 1 to 1.5 h. This extended amount of time was necessary to allow participants to fully understand and process each question before responding or getting assistance from a parent or guardian. Some participants struggled to pay attention, especially in the later portion of the CAPE administration. It may be helpful to offer a break to some children, in order to refocus and answer each question as accurately as possible.

Although the interview-administered CAPE was directed to the youth participant, parental assistance was often helpful [see Bogner (47), for an interesting review]. Discrepancies between the youth participant and their parent occasionally existed when estimating intensity, with whom they participated, or where the activity took place the most often. From the researcher’s perspective it appeared that parental estimates showed more accuracy. With a parental prompt, the youth participant usually recalled what the parent suggested. For example, when the children were asked how often they visited with others, the parents might suggest specific people with whom the child typically visited, to prompt accuracy in determining the frequency of visits.

The protocol for administering the CAPE requires the participants to establish a 4-month reference period. This was difficult for some of the participants to comprehend, and parental assistance was often required. The visual cue cards provided pictures that were helpful in describing some of the activities, but at times further explanation for both the youth and parents was necessary. A good CAPE administrator must be prepared to make items more concrete. For example, clarification was needed for the activities titled “hanging out,” “visiting,” and “entertaining others.” The level of intellectual disability appeared to influence a child’s ability to self-report. Intellect was not formally measured for the purpose of this study, but it was evident that some youth were more capable of completing the questionnaire independently, while others needed more assistance from their parents.

### Future Direction

Since many participants received help from their parents during the administration of the CAPE, it might be interesting to compare parental report to youth self-report to further verify the reliability of the CAPE for youth with DS. Parents of youth with DS appear to know a lot about what their children participate in, where they participate, and with whom they participate with.

The lengthy administration time of the interview caused difficulty in sustaining attention for some of the youth. An alternative study might focus on a subset of the questions from the domains of the most interest. Decreasing the timeframe necessary to administer this participation tool would be helpful in sustaining attention for the duration of the questionnaire.

Future research might also investigate participation engagement over time. Which activities do children continue, and which activities are dropped? A longitudinal analysis might be helpful in better understanding how participation in different activity types changes over time as well as contribute to the long-term participation patterns of children with DS.

## Conclusion

Children with DS participate in all activity types represented in the CAPE. Based on the results of this study, different activity types support different priorities. Children with DS participated the most in recreational activities. Physical and social activities allowed the greatest geographic extension into the community. Social activities involved the greatest social extension into the community. Proportionately, children with DS participate more in informal compared to formal activities. Consistent with Menear (26), the findings of this study support the need for a variety of community-based programs for youth with DS. Understanding the activity preferences of individual with DS can potentially aid parents, educators, and allied health professional in identifying attractive, meaningful, and motivating activities.

## Ethics Statement

All participants gave verbal and written assent, and a caregiver for each participant signed informed consent. All procedures were approved by the Institutional Review Board at the University of Michigan.

## Author Contributions

All the authors contributed to this manuscript. MM conceived the idea, methodology, data analysis, and interpreted results. JL contributed to the section of the manuscript focused on informal and formal activities. PE assisted in interpreting results. NC updated this manuscript with relevant literature. DU mentored MM throughout this process.

## Conflict of Interest Statement

The authors declare that the research was conducted in the absence of any commercial or financial relationships that could be construed as a potential conflict of interest.
